# Marathon-Induced Cardiac Strain as Model for the Evaluation of Diagnostic microRNAs for Acute Myocardial Infarction

**DOI:** 10.3390/jcm11010005

**Published:** 2021-12-21

**Authors:** Omid Shirvani Samani, Johannes Scherr, Elham Kayvanpour, Jan Haas, David H. Lehmann, Weng-Tein Gi, Karen S. Frese, Rouven Nietsch, Tobias Fehlmann, Steffi Sandke, Tanja Weis, Andreas Keller, Hugo A. Katus, Martin Halle, Norbert Frey, Benjamin Meder, Farbod Sedaghat-Hamedani

**Affiliations:** 1 Department of Internal Medicine III, Heidelberg University Hospital, Heidelberg University, 69120 Heidelberg, Germany; Omid.Shirvani@ukbonn.de (O.S.S.); Elham.Kayvanpour@med.uni-heidelberg.de (E.K.); Jan.Haas@med.uni-heidelberg.de (J.H.); DavidHermann.Lehmann@med.uni-heidelberg.de (D.H.L.); giwengtein@gmail.com (W.-T.G.); Karen.Frese@med.uni-heidelberg.de (K.S.F.); Rouven.Nietsch@med.uni-heidelberg.de (R.N.); steffi.sandke@med.uni-heidelberg.de (S.S.); tanja.weis@med.uni-heidelberg.de (T.W.); hugo.katus@med.uni-heidelberg.de (H.A.K.); sekretariat.frey@med.uni-heidelberg.de (N.F.); Farbod.Sedaghat-Hamedani@med.uni-heidelberg.de (F.S.-H.); 2 DZHK (German Centre for Cardiovascular Research), 69120 Heidelberg, Germany; 3 Department of Prevention, Rehabilitation and Sports Medicine, Klinikum Rechts der Isar, Technical University of Munich, 80992 Munich, Germany; Johannes.Scherr@balgrist.ch (J.S.); halle@tum.de (M.H.); 4 University Center for Prevention and Sports Medicine, Balgrist University Hospital, University of Zurich, 8008 Zurich, Switzerland; 5 Department of Clinical Bioinformatics, Saarland University, 66123 Saarbrücken, Germany; tobias.fehlmann@ccb.uni-saarland.de (T.F.); andreas.keller@ccb.uni-saarland.de (A.K.)

**Keywords:** biomarker, troponin, marathon running, microRNAs, myocardial infarction, exercise

## Abstract

Background: The current gold standard biomarker for myocardial infarction (MI), cardiac troponin (cTn), is recognized for its high sensitivity and organ specificity; however, it lacks diagnostic specificity. Numerous studies have introduced circulating microRNAs as potential biomarkers for MI. This study investigates the MI-specificity of these serum microRNAs by investigating myocardial stress/injury due to strenuous exercise. Methods: MicroRNA biomarkers were retrieved by comprehensive review of 109 publications on diagnostic serum microRNAs for MI. MicroRNA levels were first measured by next-generation sequencing in pooled sera from runners (*n* = 46) before and after conducting a full competitive marathon. Hereafter, reverse transcription quantitative real-time PCR (qPCR) of 10 selected serum microRNAs in 210 marathon runners was performed (>10,000 qPCR measurements). Results: 27 potential diagnostic microRNA for MI were retrieved by the literature review. Eight microRNAs (miR-1-3p, miR-21-5p, miR-26a-5p, miR-122-5p, miR-133a-3p, miR-142-5p, miR-191-5p, miR-486-3p) showed positive correlations with cTnT in marathon runners, whereas two miRNAs (miR-134-5p and miR-499a-5p) showed no correlations. Upregulation of miR-133a-3p (*p* = 0.03) and miR-142-5p (*p* = 0.01) went along with elevated cTnT after marathon. Conclusion: Some MI-associated microRNAs (e.g., miR-133a-3p and miR-142-5p) have similar kinetics under strenuous exercise and MI as compared to cTnT, which suggests that their diagnostic specificity could be limited. In contrast, several MI-associated microRNAs (miR-26a-5p, miR-134-5p, miR-191-5p) showed different release behavior; hence, combining cTnT with these microRNAs within a multi-marker strategy may add diagnostic accuracy in MI.

## 1. Introduction

Myocardial infarction (MI) is a relevant world-wide medical burden, which is associated with high mortality [1] and reduced quality of life in many survivors [2]. Due to its life-threatening consequences, MI needs to be diagnosed accurately and as early as possible. The current gold standard biomarker for MI is measurement of cardiac troponins (cTn), which is highly sensitive for myocardial injury and highly specific for cardiomyocytes [3,4]. However, cTn has—as single point measurement—a poor diagnostic-specificity since cTn blood concentration can be elevated in diverse cardiac [5,6,7] or non-cardiac diseases [8] as well as under physiological conditions [9]. For instance, strenuous exercise, such as marathon running, can lead to considerably elevated cTn levels [10,11] and it is still discussed controversially whether this is due to cardiomyocyte stress or cell death [11,12,13,14]. Hence, due to cTn’s lack of diagnostic specificity, the accurate selection of patients with MI remains challenging. This is particularly challenging in patients who experience corresponding clinical symptoms during or shortly after strenuous exercise.

Various novel biomarkers for MI have been investigated. Among others, these include copeptin [15], heart-type fatty acid-binding protein [16], ischemia-modified albumin [17], and microRNAs (miRNA) [18]. MiRNAs are small non-protein coding single-stranded ribonucleic acids (RNA) with a length of approximately 22 nucleotides [19]. Their main function is post-transcriptional gene regulation [20,21]. MiRNAs are not only intracellularly located, but also released into the extracellular space by different mechanisms [22]. Hence, they can be detected in various body fluids, such as blood [18], urine [23], and breast milk [24]. MiRNAs are involved in many physiological processes [25,26]. Furthermore, miRNAs are dysregulated in nearly all medical conditions [27]. Therefore, they have been investigated as potential biomarkers in many indications, including MI [18,28,29]. In this context, they have been discussed as single biomarkers [30], as signatures consisting of multiple miRNAs [18], and as part of multi-marker strategies consisting of different molecular types of biomarkers [29]. Nevertheless, similar to cTn, some potential diagnostic serum miRNAs for MI lack diagnostic-specificity [31]. Furthermore, some studies have questioned their diagnostic ability and superiority compared to conventional cardiac biomarkers [32,33]. Therefore, their clinical implementation as single miRNA assays still appears uncertain.

The aim of this study was to evaluate the specificity of potential diagnostic serum miRNAs for MI. We chose an indirect approach, by assessing miRNAs in definitely non-MI probands undergoing strenuous exercise by marathon running. We hypothesized that miRNAs that show similar kinetics in MI and cTn-increasing exercise most likely do not provide advances in diagnostic specificity. Inversely, miRNAs with differing behavior might be interesting candidates that are providing independent “biological” information. To the best of our knowledge, this is the first study systematically evaluating the specificity of potential diagnostic serum miRNAs for MI in a human in vivo model of cardiac strain by employing next-generation sequencing (NGS) and quantitative real-time polymerase chain reaction (qPCR).

## 2. Materials and Methods

### 2.1. Comprehensive Review of Potential Diagnostic Serum microRNAs as Biomarkers for Myocardial Infarction

**Figure 1:**
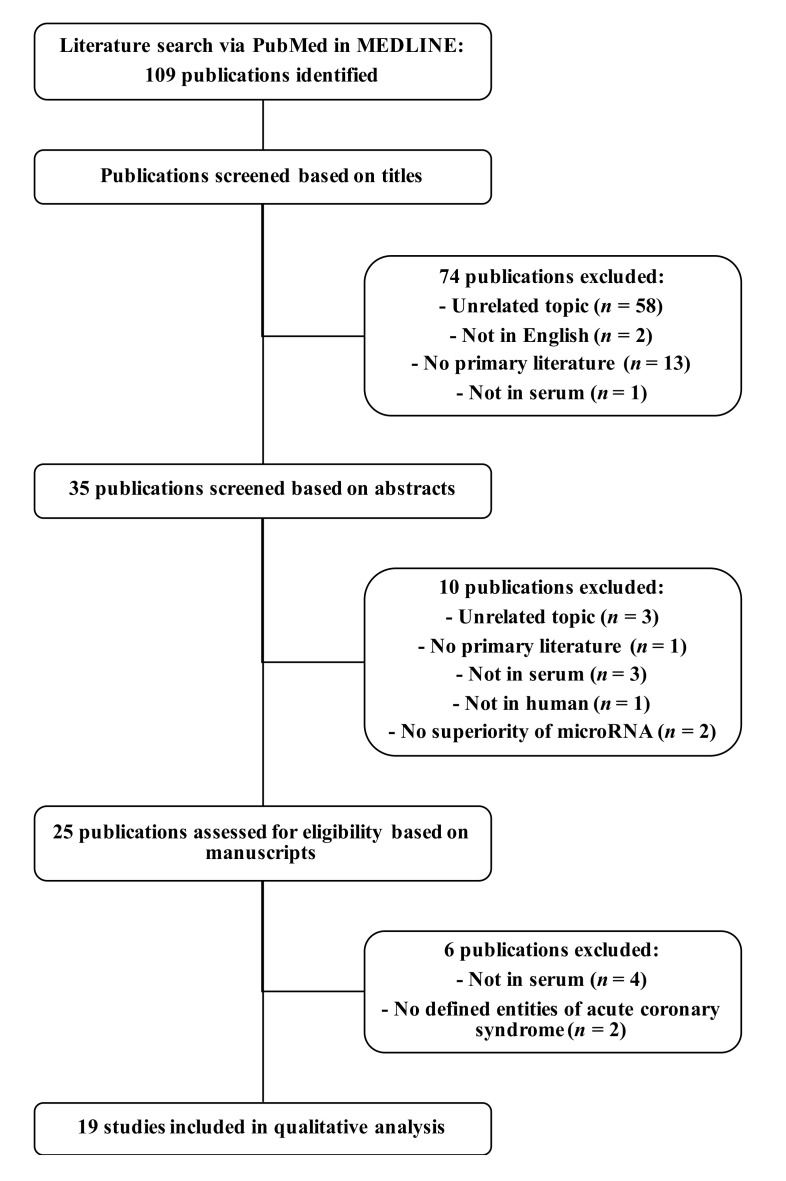
Workflow of the literature review about potential diagnostic serum microRNAs for myocardial infarction. The literature search was conducted via PubMed in the MEDLINE database. From 109 publications, 19 articles were identified presenting potential diagnostic serum microRNAs for myocardial infarction in human. Abbreviation: *n*, number of publications.

We ascertained potential diagnostic miRNAs from English-language research articles that discussed miRNAs in human serum as biomarkers for MI. The literature search was conducted via PubMed in the MEDLINE database, lastly updated on third of December 2020. We defined a search query consisting of “Medical Subject Headings” terms and keywords (Supplementary method section). Hereby, we identified 109 publications. These publications were screened based on titles and abstracts, whereupon the manuscripts were assessed for eligibility (Figure 1). Publications were excluded if not presenting miRNAs in human serum as potential diagnostic biomarkers for MI as primary literature. We did not include articles investigating miRNAs in whole blood and isolated cells. Even though circulating miRNA expressions show overlap in serum and plasma, they also present crucial differences, wherefore we only focused on serum as a blood compartment [34]. Serum miRNAs show less variability and higher sensitivity in cardiovascular diseases [34]. Finally, 19 articles remained. Where necessary, published miRNA names were adjusted to the current terminology according to the miRCarta database version 1.1 (Saarland University, Saarbrücken, Germany) [35].

### 2.2. Participants

Test persons in this study were marathon runners, who participated in the Munich-marathon in 2009. They were recruited in the context of the “Be-MaGIC“-study (Beer, Marathon, Genetics, Inflammation and the Cardiovascular system) at the Department of Prevention, Rehabilitation and Sports Medicine at the University Hospital Klinikum rechts der Isar (Technical University of Munich) in Germany. Details about the “Be-MaGIC”-study, participants characteristics’ and measurements were described previously [14,36]. Study enrollment criteria for inclusion and exclusion of participants are also provided in the Supplementary method section. Blood samples utilized in our study were taken during the final week before the marathon in fasted state as well as during the first hour after the marathon not in fasted state. Sera were separated from blood samples and stored at −80 °C within one hour. None of the samples showed visual signs of hemolysis. Serum samples of 223 marathon runners were provided for this study. According to the marathon runners’ cTnT serum concentrations (high-sensitive cTnT) after the marathon, the participants were allocated into two cohorts: the low cTnT cohort (<14 ng/L) and the high cTnT cohort (>50 ng/L). The measurements of cTnT as well as NT-proBNP from serum were conducted via a cobas e 411 analyzer (Roche Diagnostics International AG, Rotkreuz, Switzerland). The Dill and Costill equation [37] was applied on cTnT concentrations from sera that were collected after the marathon in order to adjust for the dehydration-/hyperhydration-effect induced by the marathon; nota bene, the low and high cTnT cohort for NGS and qPCR differ due to additional inclusion/exclusion criteria for participants and group-matching (details are provided in the upcoming material and method sections). In total, 46 participants were included for NGS and 210 participants were included for qPCR analyzes.

### 2.3. Ethics and Consents

Ethic committee approval was received by the ethics committee of the TUM School of Medicine at the Technical University of Munich in 2009. The approval reference number is 2384/09. Written informed consent from marathon runners were received before they participated in the trial. The study is in keeping with the ethical principles of the Declaration of Helsinki.

### 2.4. Sample Preparation for Next-Generation Sequencing

The low cTnT cohort and high cTnT cohort were matched according to age, body mass index and running time. Pooled samples, each compromising 400 µL serum, were mixed for both cohorts and each point of time: low cTnT cohort before the marathon, low cTnT cohort after the marathon, high cTnT cohort before the marathon, and high cTnT cohort after the marathon. Sample pooling was used in the first step to reduce analytical complexity and costs. Total RNA including miRNA was extracted by means of the mirVana™ PARIS™ Kit (Thermo Fisher Scientific Inc., Waltham, MA, USA). During the organic extraction (chapter E in manufacturers’ protocol), we extracted approx. 300 µL of the aqueous phase of the lysate. Hereafter, we repeated the extraction of a second aqueous phase from the same samples by adding the same amount of nuclease-free water (V = approx. 300 µL) to the remaining non-aqueous phases and repeating vortexing as well as centrifugation. For the final RNA isolation, manufacturers’ protocol’s total RNA isolation procedure was performed. Finally, the RNA was eluted in 200 µL Elution Solution. We performed an ethanol precipitation of RNA with ammonium acetate in order to reduce the volume to 12 µL (details provided in the Supplementary method section). Hereafter, 5 µL were used for library preparation by means of the TruSeq^®^ Small RNA Library Prep Kit (Illumina, San Diego, CA, USA). Quality and quantity of libraries were controlled in an Agilent 2100 Bioanalyzer system (Agilent, Santa Clara, CA, USA) utilizing the High Sensitivity DNA Kit (Agilent, Santa Clara, CA, USA). After library preparation, samples were clustered in a cBot System (Illumina, San Diego, CA, USA) using the TruSeq PE Cluster Kit v3-cBot-HS (Illumina, San Diego, CA, USA). Hereafter, short read sequencing was performed in a HiSeq^TM^ 2000 sequencing system (Illumina, San Diego, CA, USA) using the TruSeq SBS Kit v3-HS (Illumina, San Diego, CA, USA). All kits were applied according to manufacturers’ protocols’ versions in May 2016.

### 2.5. Next-Generation Sequencing: Data Preparation for Analyzes

Demultiplexing and individual base call to FASTQ conversion was performed by means of CASAVA (version 1.8.2) (Illumina, Inc., San Diego, CA, USA). MiRMaster (version 1.0) (Saarland University, Saarbrücken, Germany) was utilized for mapping, quantification, and normalization of miRNA data in 2016 [38]. Normalization was performed based on reads per million mapped reads (RPM). The Dill and Costill equation [37] was applied on miRNA expressions from sera that were collected after the marathon in order to adjust for the dehydration-/hyperhydration-effect through the marathon. Since we were handling pooled samples for NGS, we used the mean of serum concentration changes for each pooled sample, based on single serum concentration changes of each marathon runner in the particular cohort. Marathon-induced fold changes of miRNAs were calculated by the following formula: RPM of a miRNA after the marathon/RPM of a miRNA before the marathon. Ratios of fold changes were calculated in order to evaluate the difference of marathon-induced miRNA dysregulations between the high and low cTnT cohort. Ratios were calculated by the following formula: fold change of a miRNA in the high cTnT cohort/fold change of a miRNA in the low cTnT cohort. Ratios were regarded as relevantly different when its value (upregulation in the high cTnT cohort) or its reciprocal value (downregulation in the high cTnT cohort) was ≥1.5. Only miRNAs were selected for the analyzes of fold changes and ratios that presented ≥10 RPM at both points of time in both cohorts already before the adjustment for the dehydration-/hyperhydration-effect according to the Dill and Costill equation.

### 2.6. Sample Preparation for Quantitative Real-Time Polymerase Chain Reaction

In contrast to NGS, group matching was not performed for qPCR in order to keep larger cohorts for statistical analyzes. The miRNeasy Serum/Plasma Advanced Kit was applied for RNA purification on 200 µL of serum of each participant. The UniSp2, UniSp4, UniSp5 RNA Spike-in mix from the RNA Spike-in Kit, for RT was added during the purification of RNA for normalization. The purification was conducted in a QIAcube (Qiagen, Venlo, Netherlands). The extracted RNA was finally eluted in a volume of 30 µL. Total RNA including miRNA was reversely transcribed by means of the miRCURY LNA RT Kit, using 1.2 µL of eluted RNA. The miRCURY LNA miRNA PCR Assays for the selected miRNAs (Supplementary Materials Table S1) and UniSp4 (for normalization) as well as the miRCURY LNA SYBR Green PCR Kit were used for qPCR. Technical duplicates were used for each assay. Nuclease-free water served as a negative control. The qPCR was conducted in an Applied Biosystems ViiA^TM^ 7 Real-Time PCR System (Thermo Fisher Scientific Inc., Waltham, MA, USA). All kits and assays were produced by Qiagen (Venlo, The Netherlands) and applied according to manufacturers’ protocols’ versions in October 2018.

### 2.7. Quantitative Real-Time Polymerase Chain Reaction: Data Preparation for Statistical Analyzes

We defined strict exclusion criteria for the measured qPCR data in order to achieve a highly reliable data set. Samples with a C_t_ > 35 PCR-cycles or a difference of >0.5 PCR-cycles between technical duplicates were excluded. A verum sample without a C_t_ > 5 PCR-cycles smaller compared to the mean C_t_ of the corresponding negative control within the same qPCR-run was excluded. Means of the remaining technical duplicates were calculated. The Dill and Costill equation [37] was applied on miRNA expressions from sera that were collected after the marathon in order to adjust for the dehydration-/hyperhydration-effect through the marathon. Participants were excluded if information required by the Dill and Costill equation were not available (*n* = 2). For normalization of miRNAs, we used the spike-in UniSp4. Normalization was performed by the formula 2^−∆Ct^, receiving the relative quantity. ∆Ct was defined as Target-C_t_–UniSp4-C_t_. Marathon-induced fold changes of relative quantities of miRNAs were calculated by the formula 2^−∆∆Ct^. ∆∆Ct was defined as relative quantity of a miRNA after the marathon—relative quantity of a miRNA before the marathon. Our data pipeline led to different numbers of samples for each miRNA for each statistical test since miRNAs were not measurable in all samples in keeping with our prerequisites. Only participants were included, whose cTnT before the marathon was <14 ng/L in order to exclude participants with cTnT concentrations above the normal range. One participant presented a cTnT concentration of 708 ng/L after the marathon, which was regarded as an outlier value. This participant was excluded for qPCR-measurements since the value was immensely higher compared to other marathon runners. Possible reasons could have been a preanalytical or analytical failure or a deviating pathophysiology of cardiac damage.

### 2.8. Statistics

Statistic tests were performed on cohorts with sample sizes of minimum five samples. Non-logarithmized and logarithmized data (natural log transformation) were assessed for normal distribution and log-normal distribution using the Shapiro–Wilk test, histograms and quantile–quantile plots.

For statistic correlation tests, we performed a natural log transformation on miRNA relative quantities after the marathon and cTnT concentrations after the marathon since the data present approximate log-normal distribution. Outlier values of logarithmized data of miRNA and cTnT were tested by means of the generalized extreme Studentized deviate test [39] and excluded. Homoscedasticity was evaluated by means of scatter plots and Bartlett’s test. Linearity was evaluated by means of scatter plots. Correlation was tested based on the Pearson’s correlation coefficient.

For comparison of central tendencies of miRNA fold changes between the low cTnT cohort and the high cTnT cohort, the two-sided Mann–Whitney *U* test was applied since data include data strongly violating normal and log-normal distribution and cohorts consist of small number of samples (max. 40 samples). For comparison of central tendencies of characteristic parameters, the two-sided Mann–Whitney *U* test, two-sided independent-samples *t*-test, and two-sided Welch’s *t*-test were applied, depending on the distribution, scedasticity, and number of data. Homoscedasticity was evaluated by means of strip plots and Bartlett’s test.

In the context of the Mann–Whitney *U* tests, the exact *p*-value was ascertained due to number of samples (5–56 samples). We defined 0.05 as the level of significance for statistical tests. For multiple testing, we performed a false discovery rate according to Benjamini and Hochberg. Unadjusted *p*-values for presented adjusted *p*-values are provided in the Supplementary Materials Section (Tables S2 and S3). *p*-values are expressed as <0.001 if they are <0.001. They are rounded to the third decimal place if they are <0.01 and ≥0.001 or if they are <0.05 and ≥0.045. Otherwise, they are rounded to the second decimal place. Group-matching between the low and high cTnT cohort for NGS was performed based on a distance metric utilizing the Euclidean distance and subsequent hierarchical clustering according to Ward’s hierarchical clustering method using the “ward.D2” method in R (The R Foundation for Statistical Computing, Vienna, Austria) [40,41]. The Mann–Whitney *U* tests and group-matching were conducted via R (version 4.0.3 for the Mann–Whitney *U* tests; version 3.2.4 for the group matching), whereas other statistical tests and visualization of data were conducted via Python (version 3.8.2) (Python Software Foundation, Beaverton, ON, USA) using the Math module as well as following libraries: NumPy, Pandas, SciPy, Matplotlib and Seaborn.

## 3. Results

### 3.1. Comprehensive Review of Serum microRNA Biomarkers of Myocardial Infarction

After review and filtering, 19 original articles were used to extract MI miRNAs that were measured in serum (Figure 1). The 27 unique miRNAs following the selection criteria are listed in Table 1. One miRNA (miR-3656), which has been published as a serum biomarker for MI, had been removed from miRBase (Version 22.1) (University of Manchester, Manchester, UK) since it most likely does not exist. Therefore, we excluded this miRNA from our analyzes. Six miRNAs have been published multiple times, with five miRNAs (miR-1-3p, miR-21-5p, miR-126-3p, miR-133b, miR-142-5p) published twice and one (miR-499a-5p) published three times in independent articles. Eighteen miRNAs were reported as upregulated and eight as downregulated in the condition of acute MI (Table 1). One miRNA (miR-126-3p) showed controversial results, with one study demonstrating upregulation and another downregulation, possibly explained by different MI-cohorts (STEMI vs. MI) and different sample types (exosomal serum miRNAs vs. whole serum miRNAs). Altogether, we created a set of MI miRNAs for further investigation in comparison to strenuous as a non-MI condition.

### 3.2. Patients Undergoing Strenuous Exercise

We examined participants before and after a marathon race and divided the cohorts in those with troponin elevation and those without. For clinical characteristics of the tested individuals see Table 2. Of note, the cohorts with troponin elevations had higher mean heart rates (161.44 bpm ± 9.78 bpm vs. 153.24 bpm ± 9.09 bpm; *p* = 0.001). Furthermore, the runners with elevated cTnT were significantly younger (38.48 yrs ± 11.25 yrs vs. 45.19 yrs ± 5.89 yrs; *p* < 0.001). None of the cohorts showed elevated NT-proBNP concentrations. The vast majority of participants did not actively smoke during the study. Furthermore, both cohorts presented with normal BMI.

### 3.3. Next-Generation Sequencing to Identify Abundant and Reliably Measurable Serum microRNAs in Marathon Runners

**Figure 2:**
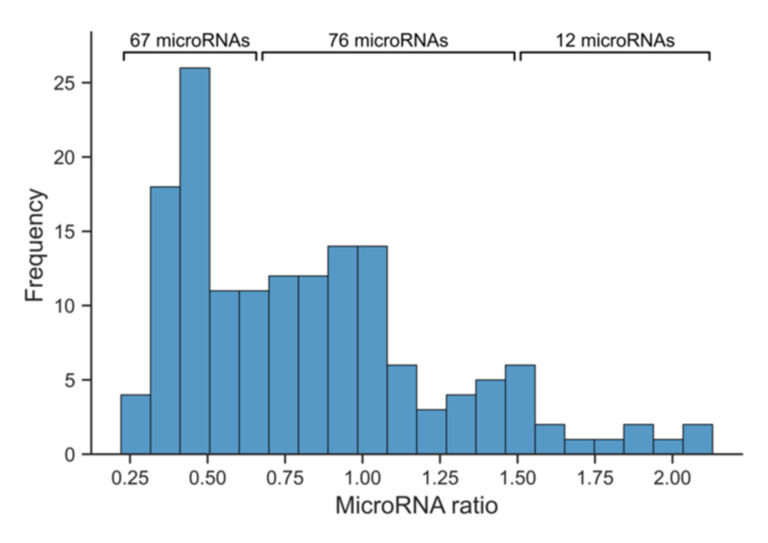
Distribution of ratios of reliably detectable microRNAs in marathon runners. The distribution of ratios of reliably detectable microRNAs in marathon runners ranges from 0.22 to 2.13. According to the ratios of these 155 microRNAs, 12 microRNAs demonstrate upregulation (cut-off value 1.5), while 67 microRNAs present downregulation (cut-off value 1/1.5) in the high cTnT cohort compared to the low cTnT cohort. In contrast, 76 microRNAs show no relevant differences of marathon-induced dysregulations between both cohorts. Ratios were calculated based on microRNA expressions from next-generation sequencing (see material and methods). Abbreviation: cTnT, cardiac troponin T.

NGS of isolated small-RNAs from pooled serum samples was conducted as a screening method, which allowed selecting miRNAs of interest that were then measured using high-sensitive qPCR. Sequencing of these samples presented abundant and reliably measurable serum miRNAs in marathon runners. The sequencing results demonstrated high numbers of sequenced reads after trimming and quality filtering with miRMaster (Supplementary Materials Table S4). Furthermore, they presented reliable ratios of mapped miRNA reads to sequenced reads after quality control (Supplementary Materials Table S4). Serum hyperhydration/dehydration during marathon accounted for 2.24–2.99% of biomarker expression alterations, as calculated by established criteria. The sequencing results demonstrated abundant miRNAs in marathon runners before and after the marathon. In total, 155 miRNAs could be reliably measured in keeping with the threshold of ≥10 RPM in the low and high cTnT cohorts at both time points. Single pooled samples showed higher numbers of detectable miRNAs (up to 198). Then, 162 miRNAs were sequenced in the low cTnT cohort before marathon, while 185 miRNAs were detected in the sample of the same cohort after marathon. Similarly, 177 miRNAs were sequenced in the high cTnT cohort before marathon, while 198 miRNAs were detected in the sample of the same cohort after marathon. In both cohorts, the number of miRNAs increased after marathon. In the low cTnT cohort it increased by 14% and in the high cTnT cohort by 12%. According to the calculated ratios of the 155 reliably measurable miRNAs in both cohorts at both time points, 12 miRNAs demonstrated upregulation, while 67 miRNAs showed downregulation in marathon runners with elevated cTnT levels compared to the low cTnT cohort (Figure 2). The remaining 76 miRNAs showed no relevant differences between the two cohorts. Concerning the potential diagnostic serum miRNAs from the literature review, the expressions of 14 of 27 miRNAs could be reliably measured in keeping with a threshold of ≥10 RPM (Supplementary Materials Table S5).

### 3.4. Quantitative Real-Time Polymerase Chain Reaction to Further Stratify Suitable Myocardial Infarction Candidate microRNAs

For statistical analyzes by means of qPCR, 10 heterogenous miRNAs (miR-1-3p, miR-21-5p, miR-26a-5p, miR-122-5p, miR-133a-3p, miR-134-5p, miR-142-5p, miR-191-5p, miR-486-3p, miR-499a-5p) were selected based on their evidence in literature and on their expression in our NGS data (Supplementary Materials Table S1). These miRNAs were published as diagnostic biomarkers of MI and included upregulated as well as downregulated miRNAs. Furthermore, this set included miRNAs presenting with same as well as with opposite dysregulation directions between marathon runners with elevated cTnT levels (based on the ratio) and patients with MI. Moreover, the selected miRNAs also included those that could not be reliably measured by NGS. In total, more than 10,000 individual qPCR measurements were conducted, and a comprehensive, high-quality data set was established based on a strict data pipeline (see material and methods) for statistical analyzes.

### 3.5. Co-Release of microRNAs and Cardiac Troponin T

**Figure 3:**
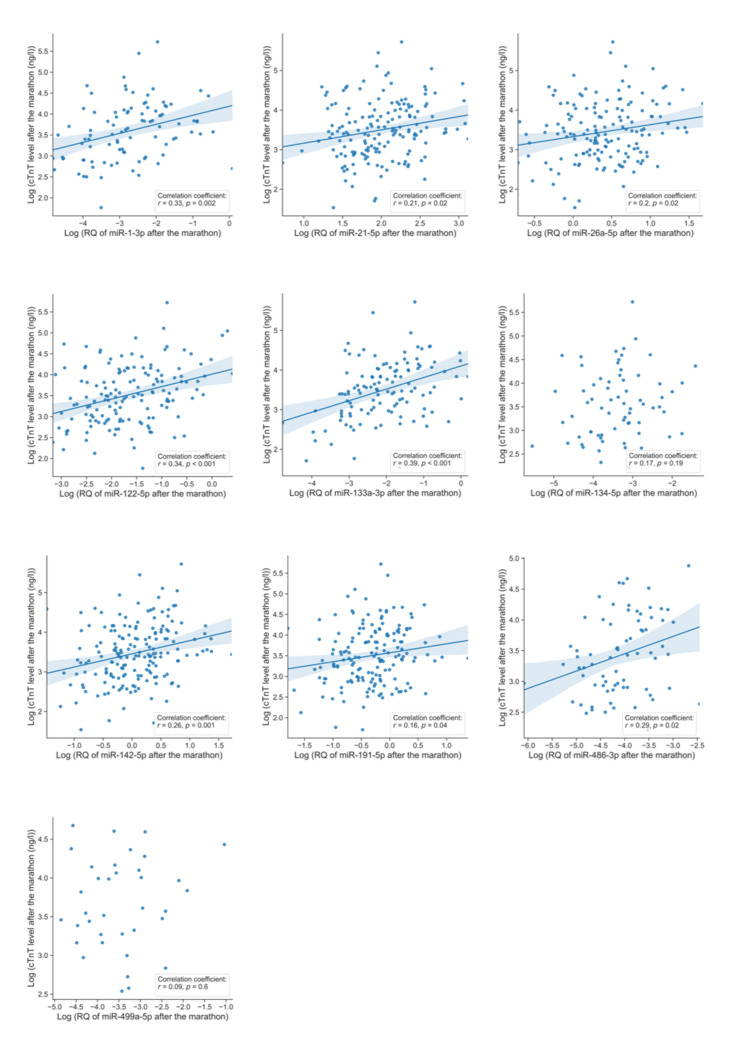
Pearson’s correlation coefficient of microRNAs and cardiac troponin T after the marathon. Pearson’s correlation coefficient was performed on relative quantities of microRNA expressions after the marathon and cTnT concentrations after the marathon of runners. Eight microRNAs present significant positive correlations. The highest correlation coefficient belongs to miR-133a-3p with *r* = 0.39. Natural log transformation was performed on data. Linear regression line and 95% confidence interval (blue area around the regression line) are plotted for significant correlations. Adjustment of *p*-values for multiple testing was performed based on false discovery rate according to Benjamini and Hochberg. MicroRNA expressions were attained by means of quantitative real-time polymerase chain reaction. Abbreviations: cTnT, cardiac troponin T; r, correlation coefficient; RQ, relative quantity.

To investigate possible co-release of miRNAs and cTnT, we tested for linear correlations of miRNA levels with cTnT levels after marathon on a per-sample basis using Pearson’s correlation on 210 individuals (Table 3, Figure 3).

**Figure 4:**
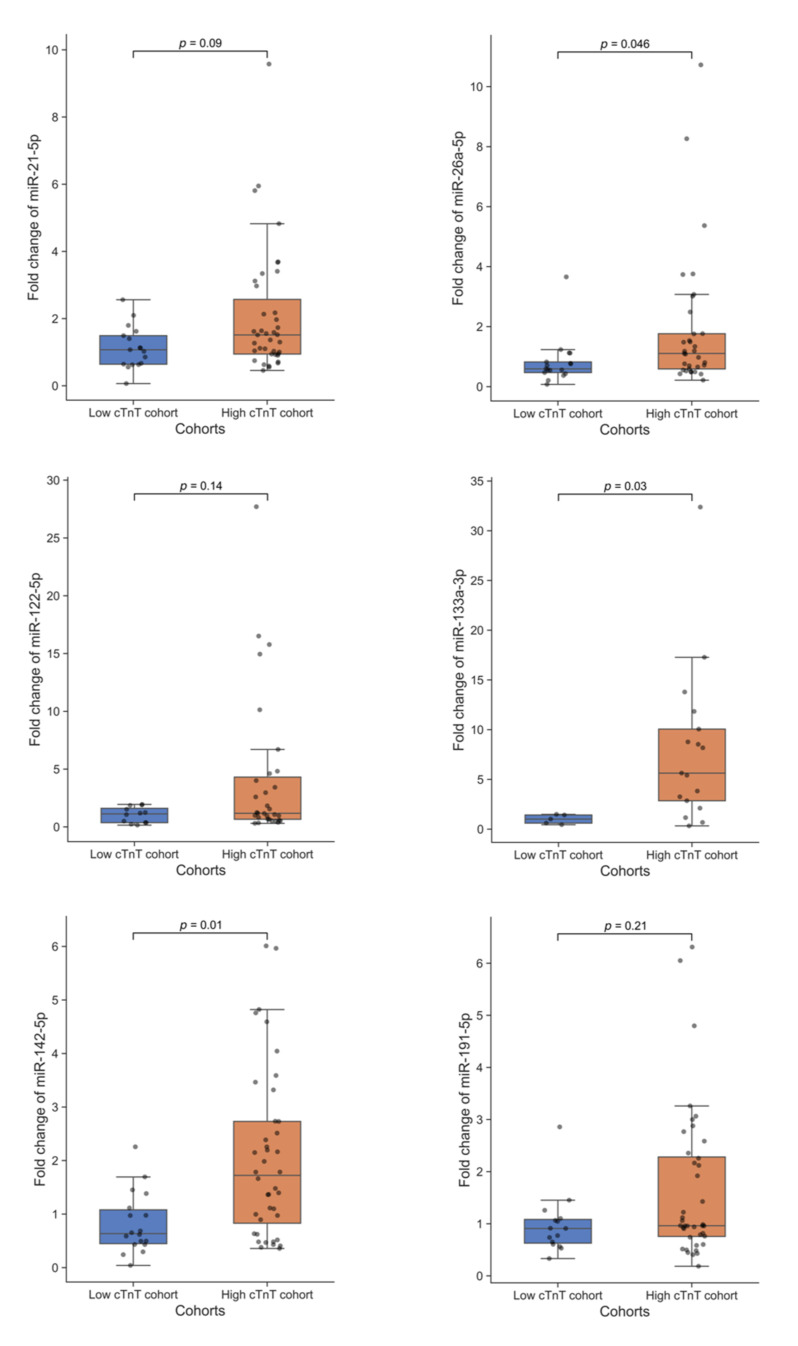
Fold change differences of microRNAs between the low and high cTnT cohort. Comparing central tendencies of microRNA fold changes between the low and high cTnT cohort, three microRNAs are upregulated in patients with elevated cTnT concentrations, none is downregulated, and three are not significantly different. The two-sided Mann–Whitney *U* test (exact *p*-value) was applied. Adjustment of *p*-values for multiple testing was performed based on false discovery rate according to Benjamini and Hochberg. MicroRNA expressions for calculation of fold changes (see material and methods) were attained by means of quantitative real-time polymerase chain reaction. Abbreviation: cTnT, cardiac troponin T.

Eight miRNAs (miR-1-3p, miR-21-5p, miR-26a-5p, miR-122-5p, miR-133a-3p, miR-142-5p, miR-191-5p, miR-486-3p) presented moderate but significant positive correlations with cTnT (r: 0.16–0.39). The highest correlation coefficient was noted for the cardiac specific miR-133a-3p (r: 0.39, *p* < 0.001). The remaining two miRNAs (miR-134-5p and miR-499a-5p) presented no significant correlations with cTnT levels after marathon. The differences of marathon-induced fold changes were then tested for statistical significance by Mann–Whitney *U* test comparing the high cTnT cohort (*n* = 56) with the low cTnT cohort (*n* = 31) (Table 4, Figure 4).

Three miRNAs (miR-26a-5p, miR-133a-3p, miR-142-5p) were significantly upregulated in marathon runners with elevated cTnT levels compared to those without elevated cTnT. From these, two miRNAs (miR-133a-3p and miR-142-5p) were dysregulated into the same direction as in patients with MI (both upregulated) and one (miR-26a-5p) was dysregulated into opposite directions (upregulated after marathon with cTnT increase, but downregulated in MI) as shown in Table 5.

## 4. Discussion

Troponin biomarkers revolutionized MI diagnosis and care. However, since they lack diagnostic specificity, several novel potential biomarkers have been proposed, including numerous miRNAs. However, superiority of single miRNA assays compared to cardiac troponin has not been successfully demonstrated. We conducted here a comprehensive literature review in order to identify serum miRNAs for MI and analyzed their specificity by means of NGS and qPCR in a non-MI setting: marathon running.

Based on our comprehensive analyzes, this study distinguishes miRNAs with similar and divergent release behavior between patients with MI and marathon runners with exercise-induced cTn elevation. MiRNAs with similar kinetics to cTn in both conditions presumably indicate myocardial stress/injury rather than being specific for the pathophysiology of MI. MiRNAs with divergent kinetics presumably present different aspects of the pathophysiology of MI and may add troponin-independent information. Hence, clinical implementation should try to combine troponin biomarkers and troponin-unrelated miRNAs.

Beside of miRNAs’ role in diagnostics, they seem to influence adaptive and maladaptive cardiac remodeling, such as myocardial hypertrophy and fibrosis [62]. Interestingly, different types of prolonged and strenuous exercise result in blood level alterations of circulating miRNAs [62]. Our results about miRNAs’ exercise-induced cTn-related kinetics may help to evolve their clinical impact on cardiovascular prevention. Hypothetically, miRNAs with similar kinetics to cTn could be predominantly involved in maladaptive cardiac pathways, since cTn elevation after exercise is associated with cardiovascular events [63]. However, further research is needed to evaluate this topic.

In respect of the ten selected miRNAs of our study, miR-1-3p, miR-21-5p, miR-122-5p, miR-133a-3p, miR-142-5p, and miR-486-3p appear to be unspecific for MI and rather reflect myocardial injury. These miRNAs have been all reported as upregulated in literature (Table 1). In our data, these miRNAs significantly and positively correlated with cTnT in marathon runners. MiR-133a-3p and miR-142-5p were even significantly upregulated in marathon runners with elevated cTnT levels compared to those without elevated cTnT after applying Mann–Whitney *U* test. Similarly to our hypothesis, other authors have reported some of these miRNAs in other conditions of myocardial injury/stress independent of MI. MiR-122-5p has been shown to be elevated in patients with chemotherapy-induced cTn raise [64]. May et al. have demonstrated miR-1-3p and miR-133a-3p to be upregulated after elective non-cardiac surgery, probably induced by myocardial stress [65]. Additionally, upregulation of both miRNAs may be involved in atrial remodeling induced by physical exercise [66]. Goldbergova et al. even concluded that both miRNAs are not diagnostically superior to cTn in patients with MI [33], questioning their potential as single biomarkers.

Three miRNAs showed promising results as possible candidates for a multi-marker strategy that adds diagnostic accuracy to cTnT in case of MI. These three miRNAs were differently regulated between MI and part of our cohort with elevated cTnT after marathon. MiR-134-5p did not correlate with marathon-induced cTnT release, while it has been reported as upregulated in MI [43]. We hypothesize that its upregulation in MI happens via mechanisms different and independent from cTnT release. MiR-26a-5p and miR-191-5p correlated positively with cTnT in runners after marathon, while they have been reported to be downregulated in MI [48]. These two miRNAs may convey a cardioprotective effect. Xing et al. suggested that miR-26-5p protects against myocardial ischemia/reperfusion injury through regulating the PTEN/PI3K/AKT signaling pathway [67]. Yang et al. also showed that both miR-26a-5p and miR-191-5p levels were lower in patients with major adverse cardiovascular events (MACE) after myocardial infarction [68]. A pathway analysis on miRPathDB v2.0 using Gene Ontology-Biological processes as a database with only strong grade of evidence showed that miR-26a-5p and miR-191-5p are both involved in cell cycle pathways (Supplementary Materials Figure S1) [69].

Our comprehensive literature review identified miR-499a-5p as the most frequently published potential diagnostic serum miRNA for MI. It is upregulated in MI [43,51,56], while it showed no correlation with cTnT after marathon in our data, which can be attributed to the low number of samples for this miRNA caused by its low expression state in serum [65]. MiR-499a-5p has already been reported as upregulated in perioperative myocardial injury [65], viral myocarditis [31], and acute heart failure [31], wherefore it appears unspecific for the pathophysiology of MI, but rather indicates cardiomyocytic stress or injury. In keeping with these findings, other authors such as M. Heokstra and Corsten et al. have stated that miR-499a-5p might represent cardiac distress [70] and damage [31]. Furthermore, miR-499a-5p has been previously described as not being superior compared to cTn as biomarker in early MI [71], which further reduces its potential as single diagnostic biomarker.

Numerous studies have addressed the question of whether miRNAs will be beneficial in diagnostics of MI. Previously, it has been shown that the utilization of machine learning methods on a high-dimensional data set of circulating miRNAs in patients with acute coronary syndromes can outreach the diagnostic accuracy of a single cTn measurement [72]. These findings emphasize that miRNAs owe the potential of improving the current strategy of MI-diagnostics, which incorporates the evaluation of serial cTn’s concentrations and their kinetic [3]. In contrast, it has been recently demonstrated that single miRNAs and a signature of best performing miRNAs can fail adding diagnostic accuracy to cTn serial measurements for detection of NSTEMI [32]. Both studies show relevant differences in terms of study design [32,72], wherefore miRNAs potential for clinical implementation still remains unclear.

In synopsis of the current literature and our study results, we recommend the utilization of multi-dimensional molecular data sets consisting of MI specific serum miRNAs plus troponin in order to receive comprehensive but independent information about the disease. As we have shown in this study, several miRNAs (miR-26a-5p, miR-134-5p, miR-191-5p) may have the ability to provide clinicians cTn independent information in the setting of MI.

For implementation of serum miRNAs into routine diagnostics, highly sensitive detection methods need to evolve. As shown in Table 3, the detection variability between miRNAs is extensive, primarily due to their extremely low and variable concentration in serum. Fortunately, there are innovative technical developments under investigation with promising improvements concerning sensitivity [73].

Potential limitations: All participants in this study were men, which hampers the generalization to females. Additionally, it cannot be excluded that certain miRNAs are also expressed in skeletal muscle and hence could have been released from tissues other than heart. Importantly, this would not interfere with the miRNAs that do not show upregulation during strenuous exercise, but in MI.

## Figures and Tables

**Table 1:** Potential diagnostic serum microRNAs for myocardial infarction from literature review.

MicroRNA	Disease *	Direction of Dysregulation	Reference
miR-1-3p	MI	Upregulation	[42,43]
miR-21-5p	ACS MI	Upregulation	[44] [45]
miR-22-5p	STEMI	Upregulation	[46]
miR-23a-3p	STEMI	Downregulation	[47]
miR-26a-5p	STEMI	Downregulation	[48]
miR-32-5p	MI	Upregulation	[49]
miR-122-5p	MI	Upregulation	[50]
miR-126-3p	MI STEMI	Upregulation Downregulation	[44] [48]
miR-133a-3p	MI	Upregulation	[51]
miR-133b	MI STEMI	Upregulation	[51] [46]
miR-134-5p	MI	Upregulation	[43]
miR-142-5p	MI	Upregulation	[52,53]
miR-145-5p	ACS	Downregulation	[54]
miR-150-3p	STEMI	Upregulation	[48]
miR-186-5p	MI	Upregulation	[43]
miR-191-5p	STEMI	Downregulation	[48]
miR-204-5p	STEMI	Downregulation	[55]
miR-208a-3p	MI	Upregulation	[43]
miR-210-3p	NSTEMI	Upregulation	[56]
miR-223-3p	MI	Upregulation	[43]
miR-363-3p	MI	Upregulation	[57]
miR-486-3p	STEMI	Upregulation	[48]
miR-492	MI	Upregulation	[58]
miR-499a-5p	MI NSTEMI	Upregulation	[43,51] [56]
miR-1915-3p	MI	Downregulation	[59]
miR-3656 **	MI	Downregulation	[59]
miR-4507	MI	Downregulation	[59]
miR-4478	NSTEMI	Downregulation	[60]

Abbreviations: ACS, acute coronary syndrome; MI, myocardial infarction; NSTEMI, non-ST-elevation myocardial infarction; STEMI, ST-elevation myocardial infarction. * “Disease” refers to the investigated study’s cohort that includes myocardial infarction or is an entity of myocardial infarction. Presented microRNAs were investigated in these cohorts as diagnostic biomarkers. ** miR-3656 has been removed from miRBase (Version 22.1) (University of Manchester, Manchester, United Kingdom) since it most likely does not exist.

**Table 2:** Characteristics of study cohorts.

Method	NGS		qPCR		
Cohort	Low cTnT Cohort (*n* = 19)	High cTnT Cohort (*n* = 27)	Low cTnT Cohort *n* = 31)	High cTnT Cohort (*n* = 56)	Correlation Cohort (*n* = 210)
Age (yrs) ($\bar{x}$ ± σ)	46 ± 5.03 (*n* = 19)	48.44 ± 6.69 (*n* = 27)	45.19 ± 5.89 (*n* = 31)	38.48 ± 11.25 * (*n* = 56)	41.63 ± 9.16 (*n* = 210)
Body-Mass-Index (kg/m2) ($\bar{x}$ ± σ)	23.18 ± 1.92 (*n* = 19)	24.19 ± 2.35 (*n* = 27)	23.39 ± 2.31 (*n* = 31)	23.25 ± 2.07 (*n* = 56)	23.55 ± 2.12 (*n* = 209)
Active smokers (*n*)	0 (*n* = 19)	0 (*n* = 27)	0 (*n* = 31)	0 (*n* = 56)	6 (*n* = 210)
Maximum heart rate ^§^ (bpm) ($\bar{x}$ ± σ)	175.8 ± 3.43 (*n* = 19)	174.09 ± 4.59 (*n* = 27)	176.36 ± 4.06 (*n* = 31)	181.06 ± 7.8 (*n* = 56)*	178.86 ± 6.4 (*n* = 210)
Running time during marathon (h:min) ($\bar{x}$ ± σ)	4:01 ± 0:32 (*n* = 19)	3:55 ± 0:32 (*n* = 27)	3:53 ± 0:30 (*n* = 26)	3:49 ± 0:31 (*n* = 53)	3:50 ± 0:30 (*n* = 196)
Mean heart rate during the marathon ^§§^ (bpm) ($\bar{x}$ ± σ)	150.94 ± 10.2 (*n* = 18)	154.91 ± 10.02 (*n* = 23)	153.24 ± 9.09 (*n* = 25)	161.44 ± 9.78 * (*n* = 43)	156.66 ± 10 (*n* = 163)
Cardiac troponin T before the marathon (ng/L) ($\hat{x}$ (Q1–Q3))	3 (3–3) (*n* = 19)	5.75 (3.91–10.13) * (*n* = 27)	3 (3–3.18) (*n* = 31)	4.15 (3–5.98) * (*n* = 56)	3 (3–4.92) (*n* = 210)
Cardiac troponin T after the marathon (ng/L) ($\hat{x}$ (Q1–Q3))	11.41 (6.36–12.72) (*n* = 19)	64.34 (58.06–89.81) * (*n* = 27)	10.98 (7.22–12.91) (*n* = 31)	67.95 (58.55–96.2) * (*n* = 56)	31.44 (18.22–53.33) (*n* = 210)
N-terminal pro-brain natriuretic peptide before the marathon (pg/mL) ($\hat{x}$ (Q1–Q3))	31.63 (21.74–54.64) (*n* = 19)	37.95 (20.12–55.02) (*n* = 27)	28.54 (18.17–38.93) (*n* = 31)	21.94 (10.38–37.58) (*n* = 56)	24.81 (13.12–42.62) (*n* = 210)

Abbreviations: bpm, beats per minute; cTnT, cardiac troponin T; *n*, number of marathon runners; NGS, next-generation sequencing; qPCR, quantitative real-time polymerase chain reaction; Q1, first quartile; Q3, third quartile; yrs, years; σ, standard deviation; $\bar{x}$, arithmetic mean; $\hat{x}$, median. ^§^ Maximum heart rate was calculated by following formula: HFmax = 208 − 0.7 × age (yrs) [61]. ^§§^ Mean heart rate during the marathon was measured by participants’ own heart rate monitors. * Labelled parameters demonstrate significant differences of central tendencies between high and low cTnT cohorts of the same quantification method (level of significance = 0.05; statistic tests used as appropriate (Mann–Whitney *U* test, independent-samples *t*-test, Welch’s *t*-test)).

**Table 3:** Pearson’s correlation coefficient for correlations between Log (relative quantity of microRNA after the marathon) * from qPCR and Log (cTnT after the marathon) *.

MicroRNA	Correlation Coefficient	*p*-Value **	Reliably Measurable in Number of Runners
miR-1-3p	r = 0.33	*p* = 0.002	*n* = 95
miR-21-5p	r = 0.21	*p* = 0.02	*n* = 163
miR-26a-5p	r = 0.2	*p* = 0.02	*n* = 151
miR-122-5p	r = 0.34	*p* < 0.001	*n* = 147
miR-133a-3p	r = 0.39	*p* < 0.001	*n* = 120
miR-134-5p	r = 0.17	*p* = 0.19	*n* = 69
miR-142-5p	r = 0.26	*p* = 0.001	*n* = 176
miR-191-5p	r = 0.16	*p* = 0.04	*n* = 167
miR-486-3p	r = 0.29	*p* = 0.02	*n* = 73
miR-499a-5p	r = 0.09	*p* = 0.6	*n* = 36

Abbreviations: cTnT, cardiac troponin T; *n*, number of marathon runners; qPCR, quantitative real-time polymerase chain reaction; r, correlation coefficient. * Natural log transformation was performed on data. ** Adjustment of *p*-values for multiple testing was performed based on false discovery rate according to Benjamini and Hochberg. Level of significance = 0.05.

**Table 4:** Difference of central tendencies of microRNA fold changes * from qPCR.

MicroRNA	Fold Change in the Low cTnT Cohort (Median, Q1–Q3)	Fold Change in the High cTnT Cohort (Median, Q1–Q3)	*p*-Value **
miR-1-3p	N/A (*n* = 1)	2.1, 1.32–4.6 (*n* = 16)	*p* = N/A ***
miR-21-5p	1.07, 0.64–1.49 (*n* = 17)	1.51, 0.95–2.57 (*n* = 39)	*p* = 0.09
miR-26a-5p	0.6, 0.47–0.82 (*n* = 17)	1.11, 0.59–1.77 (*n* = 33)	*p* = 0.046
miR-122-5p	1.13, 0.37–1.61 (*n* = 12)	1.19, 0.66–4.31 (*n* = 31)	*p* = 0.14
miR-133a-3p	1.01, 0.61–1.43 (*n* = 5)	5.63, 2.86–10.06 (*n* = 17)	*p* = 0.03
miR-134-5p	N/A (*n* = 1)	2.69, 2.14–3.13 (*n* = 5)	*p* = N/A
miR-142-5p	0.64, 0.45–1.08 (*n* = 18)	1.72, 0.83–2.73 (*n* = 40)	*p* = 0.01
miR-191-5p	0.91, 0.63–1.08 (*n* = 15)	0.96, 0.76–2.28 (*n* = 40)	*p* = 0.21
miR-486-3p	N/A (*n* = 4)	N/A (*n* = 4)	*p* = N/A
miR-499a-5p	N/A (*n* = 0)	N/A (*n* = 1)	*p* = N/A

Abbreviations: cTnT, cardiac troponin T; *n*, number of marathon runners; N/A, not applicable; Q1, first quartile; Q3, third quartile; qPCR, quantitative real-time polymerase chain reaction. * Fold change is defined as 2^−∆∆Ct^ (see materials and methods). ** The two-sided Mann–Whitney *U* test (exact *p*-value) was applied for comparison of central tendencies of microRNA fold changes between the low cTnT cohort and the high cTnT cohort. Adjustment of *p*-values for multiple testing was performed based on false discovery rate according to Benjamini and Hochberg. Level of significance = 0.05. *** N/A: Four of the 10 microRNAs (miR-1-3p, miR-134-5p, miR-486-3p, miR-499a-5p) could not be included in this statistic test due to small number of reliably measured data points (very low abundance microRNAs).

**Table 5:** Directions of microRNA dysregulation of patients with myocardial infarction and marathon runners.

MicroRNA	Direction of Dysregulation in Patients with MI *	Direction of Dysregulation in Marathon Runners with cTnT Rise from qPCR **
miR-1-3p	Upregulation	N/A
miR-21-5p	Upregulation	No significant difference of Dysregulation ***
miR-22-5p	Upregulation	N/A
miR-23a-3p	Downregulation	N/A
miR-26a-5p	Downregulation	Upregulation
miR-32-5p	Upregulation	N/A
miR-122-5p	Upregulation	No significant difference of dysregulation
miR-126-3p	Upregulation and downregulation	N/A
miR-133a-3p	Upregulation	Upregulation
miR-133b	Upregulation	N/A
miR-134-5p	Upregulation	N/A
miR-142-5p	Upregulation	Upregulation
miR-145-5p	Downregulation	N/A
miR-150-3p	Upregulation	N/A
miR-186-5p	Upregulation	N/A
miR-191-5p	Downregulation	No significant difference of dysregulation
miR-204-5p	Downregulation	N/A
miR-208a-3p	Upregulation	N/A
miR-210-3p	Upregulation	N/A
miR-223-3p	Upregulation	N/A
miR-363-3p	Upregulation	N/A
miR-486-3p	Upregulation	N/A
miR-492	Upregulation	N/A
miR-499a-5p	Upregulation	N/A
miR-1915-3p	Downregulation	N/A
miR-4507	Downregulation	N/A
miR-4478	Downregulation	N/A

Abbreviations: cTnT, cardiac troponin T; MI, myocardial infarction; N/A, not applicable; qPCR, quantitative real-time polymerase chain reaction. * Direction of dysregulation in patients with MI is ascertained from our literature review. ** Direction of dysregulation in marathon runners with cTnT rise from qPCR is based on differences of central tendencies of microRNA fold changes (Table 4). *** No significant difference of dysregulation means that there is no significant difference of central tendencies of microRNA fold changes between the low and high cTnT cohorts tested by the Mann–Whitney *U* test (Table 4).

## Data Availability

Not applicable.

## Supplementary Materials

The following are available online at https://www.mdpi.com/article/10.3390/jcm11010005/s1, Figure S1: Pathway analysis of miR-26a-5p and miR-191-5p, Table S1: MicroRNAs for qPCR, Table S2: Unadjusted *p*-values of the two-sided Mann–Whitney U test for comparison of central tendencies of microRNA fold changes from qPCR, Table S3: Unadjusted *p*-values of Pearson’s correlation coefficient for correlations between Log (relative quantity of microRNA after the marathon) from qPCR and Log (cTnT after the marathon), Table S4: Reads from NGS, Table S5: Expressions of reliably detectable potential diagnostic serum microRNAs in RPM from NGS. Supplementary method section: Search query, Study enrollment criteria and Precipitation of RNA.
