# Supplementary material for: Marathon-Induced Cardiac Strain as Model for the Evaluation of Diagnostic microRNAs for Acute Myocardial Infarction
Source: J Clin Med. 2021 Dec 21;11(1):5. doi: 10.3390/jcm11010005 (PMC8745173; doi:10.3390/jcm11010005)
Supplement: Supplementary file 1 [file jcm-11-00005-s001.zip › jcm-1496413-supplementary.pdf]

Supplementary Tables

Table S1: MicroRNAs for qPCR.

MicroRNA	Accession*	Nucleic acid sequence*
hsa-miR-1-3p	MIMAT0000416	5'-UGGAAUGUAAAGAAGUAUGUAU-3'
hsa-miR-21-5p	MIMAT0000076	5'-UAGCUUAUCAGACUGAUGUUGA-3'
hsa-miR-26a-5p	MIMAT0000082	5'-UUCAAGUAAUCCAGGAUAGGCU-3'
hsa-miR-122-5p	MIMAT0000421	5'-UGGAGUGUGACAAUGGUGUUUG-3'
hsa-miR-133a-3p	MIMAT0000427	5'-UUUGGUCCCCUUCAACCAGCUG-3'
hsa-miR-134-5p	MIMAT0000447	5'-UGUGACUGGUUGACCAGAGGGG-3'
hsa-miR-142-5p	MIMAT0000433	5'-CAUAAAGUAGAAAGCACUACU-3'
hsa-miR-191-5p	MIMAT0000440	5'-CAACGGAAUCCCAAAAGCAGCUG-3'
hsa-miR-486-3p	MIMAT0004762	5'-CGGGGCAGCUCAGUACAGGAU-3'
hsa-miR-499a-5p	MIMAT0002870	5'-UUAAGACUUGCAGUGAUGUUU-3'

Abbreviations: A, adenosine; C, cytidine; G, guanosine; qPCR, quantitative real-time polymerase chain reaction; U, uridine.
* Accession and nucleic acid sequence were ascertained from miRBase (version 22.1).

Table S2: Unadjusted p-values of the two-sided Mann–Whitney *U* test for comparison of central tendencies of microRNA fold changes* from qPCR.

MicroRNA	*P*-value**
miR-1-3p	p = *N*/A
miR-21-5p	$p = 0.06$
miR-26a-5p	$p = 0.02$
miR-122-5p	$p = 0.12$
miR-133a-3p	$p = 0.009$
miR-134-5p	p = *N*/A
miR-142-5p	$p = 0.002$
miR-191-5p	$p = 0.21$
miR-486-3p	p = *N*/A
miR-499a-5p	p = *N*/A

Abbreviations: cTnT, cardiac troponin T; N/A, not applicable; qPCR, quantitative real-time polymerase chain reaction.
* Fold change is defined as $2^{-\Delta\Delta Ct}$ (see materials and methods). ** The two-sided Mann–Whitney *U* test (exact *p*-value) was applied for comparison of central tendencies of microRNA fold changes between the low cTnT cohort and the high cTnT cohort.

Table S3: Unadjusted p-values of Pearson's correlation coefficient for correlations between Log (relative quantity of microRNA after the marathon)* from qPCR and Log (cTnT after the marathon)*.

MicroRNA	P-Value
miR-1-3p	$p < 0.001$
miR-21-5p	$p = 0.008$
miR-26a-5p	$p = 0.02$
miR-122-5p	$p < 0.001$
miR-133a-3p	$p < 0.001$
miR-134-5p	$p = 0.17$
miR-142-5p	$p < 0.001$
miR-191-5p	$p= 0.04$
miR-486-3p	$p = 0.01$
miR-499a-5p	$p = 0.6$

Abbreviations: cTnT, cardiac troponin T; qPCR, quantitative real-time polymerase chain reaction.
* Natural log transformation was performed on data.

Table S4: Reads from NGS.

Type of reads	Low cTnT cohort before the marathon	High cTnT cohort before the marathon	Low cTnT cohort after the marathon	High cTnT cohort after the marathon
Sequenced reads	14994008	15542241	18738749	11794043
After trimming and quality filtering	6365477	7826242	12566742	5386916
Mapping to genome	1535240	2817076	3944186	1651037
Mapping to microRNAs	1474825	2720492	3828613	1660174

Abbreviations: cTnT, cardiac troponin T; NGS, next-generation sequencing.

Table S5: Expressions of reliably detectable potential diagnostic serum microRNAs in RPM from NGS.

MicroRNA	Low cTnT cohort before the marathon	High cTnT cohort before the marathon	Low cTnT cohort after the marathon	High cTnT cohort after the marathon
miR-21-5p	1193	2856.55	2642.71	2284.21
miR-23a-3p	98.5	152.04	246.18	404.85
miR-26a-5p	1293.54	3158.95	1992.12	2110.72
miR-122-5p	54.67	56.43	41.45	44.16
miR-126-3p	71.01	165.4	148.22	150.89
miR-133a-3p	12.1	16.52	49.1	59.57
miR-142-5p	747	1497.33	1738.29	2417.07
miR-186-5p	476.63	635.06	662.88	942.95
miR-191-5p	1383.09	1818.48	978.23	1532.38
miR-204-5p	10.21	13.18	21.46	29.92
miR-210-3p	73.68	66.83	74.73	111.7
miR-223-3p	140.29	266.57	425.27	576.77
miR-363-3p	78.71	143.13	127.45	213.85
miR-486-3p	56.71	80.57	55.43	38.02

Abbreviations: cTnT, cardiac troponin T; NGS, next-generation sequencing; RPM, reads per million mapped reads.

Supplementary Figure

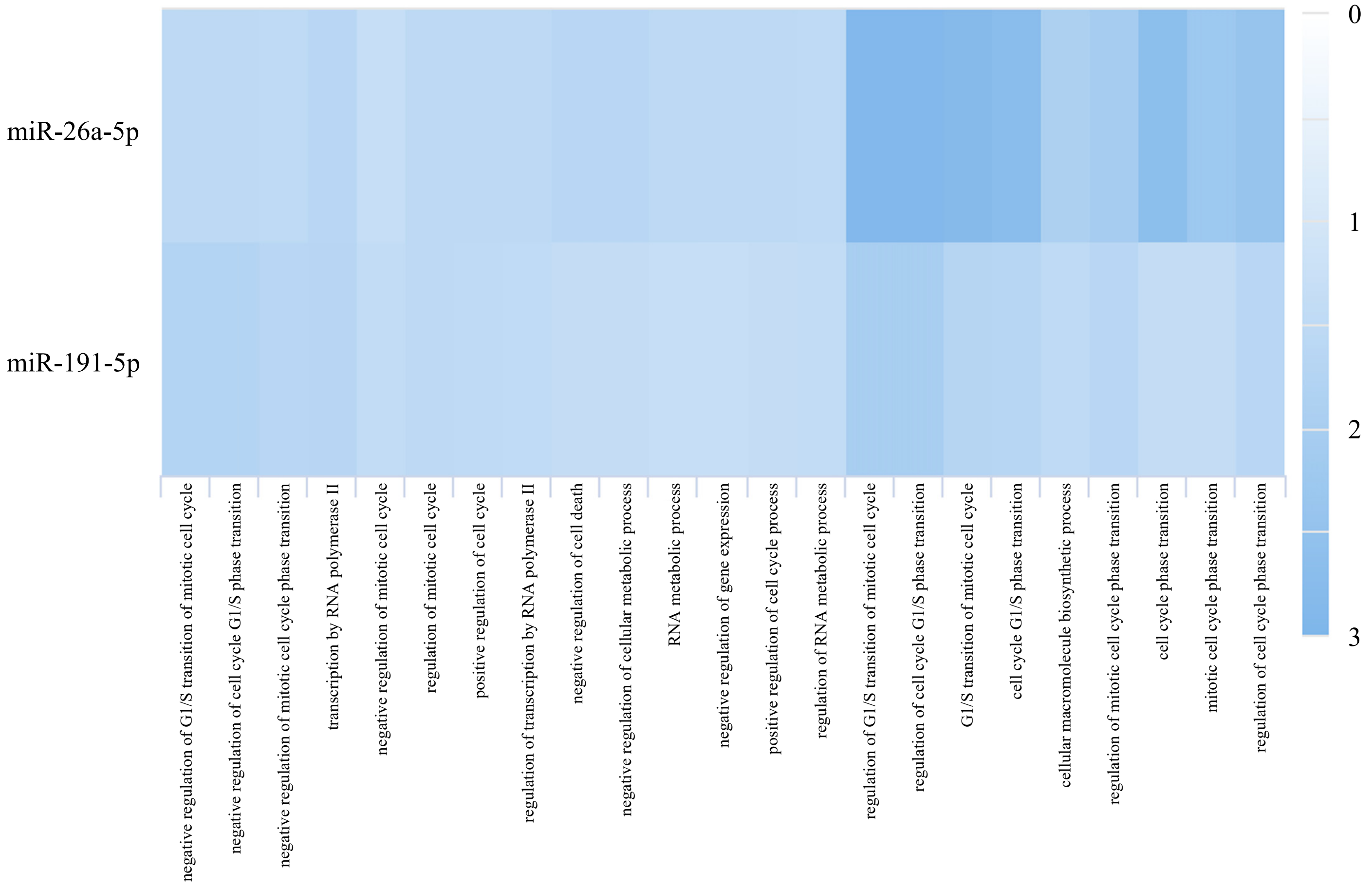

Figure S1. Pathway analysis of miR-26a-5p and miR-191-5p.

The heatmap was created by highcharts.com via miRPathDB v2.0 and modified by us. The heatmap presents Gene Ontology terms concerning miR-26a-5p (first row) and miR-191-5p (second row) based on Gene Ontology-Biological processes as database with a strong grade of evidence. Both microRNAs demonstrate involvements in cell cycle pathways. The darker the blue field, the more significant associations between the presented microRNA and pathway exist. For more details, please see the publication by Kehl et al. (PMID: 31691816). Gene Ontology terms from left to right: 1. negative regulation of G1/S transition of mitotic cell cycle, 2. negative regulation of cell cycle G1/S phase transition, 3. negative regulation of mitotic cell cycle phase transition, 4. transcription by RNA polymerase II, 5. negative regulation of mitotic cell cycle, 6. regulation of mitotic cell cycle, 7. positive regulation of cell cycle, 8. regulation of transcription by RNA polymerase II, 9. negative regulation of cell death, 10. negative regulation of cellular metabolic process, 11. RNA metabolic process, 12. negative regulation of gene expression, 13. positive regulation of cell cycle process, 14. regulation of RNA metabolic process, 15. regulation of G1/S transition of mitotic cell cycle, 16. regulation of cell cycle G1/S phase transition, 17. G1/S transition of mitotic cell cycle, 18. cell cycle G1/S phase transition, 19. cellular macromolecule biosynthetic process, 20. regulation of mitotic cell cycle phase transition, 21. cell cycle phase transition, 22. mitotic cell cycle phase transition, 23. regulation of cell cycle phase transition.

Supplementary Method Section

Search query:

("Acute Coronary Syndrome"[MeSH Terms] OR "Myocardial Infarction"[MeSH Terms] OR "acute coronary syndrome*"[Title/Abstract] OR "myocardial infarction*"[Title/Abstract] OR "st elevation myocardial infarction*"[Title/Abstract] OR "non st elevation myocardial infarction*"[Title/Abstract] OR "heart attack*"[Title/Abstract] OR "ACS"[Title/Abstract] OR "ACSs"[Title/Abstract] OR "AMI"[Title/Abstract] OR "AMIs"[Title/Abstract] OR "MI"[Title/Abstract] OR "MIs"[Title/Abstract] OR "stemi*"[Title/Abstract] OR "nstemi*"[Title/Abstract]) AND ("MicroRNAs"[MeSH Terms] OR "microrna*"[Title/Abstract] OR "mirna*"[Title/Abstract] OR "miR"[Title/Abstract] OR "miRs"[Title/Abstract]) AND ("Biomarkers"[MeSH Terms] OR "biomarker*"[Text Word] OR "marker*"[Text Word]) AND ("Diagnosis"[MeSH Terms] OR "diagnos*"[Text Word] OR "diagnostic*"[Text Word]) AND ("Serum"[MeSH Terms] OR "serum*"[Text Word] OR "sera*"[Text Word])

Study enrollment criteria

Inclusion criteria:

- Male sex
- Age between 20 - 60 years
- At least one half-marathon successfully performed in the past
- Aim to take part at the Munich-Marathon 2009 with a distance of 42,195 km
- Written informed consent

Exclusion criteria:

- Cardiac, musculoskeletal or psychiatric disease
- Neoplasia or malabsorption
- Acute or chronic infection

- Acute or chronic inflammatory disease
- Medication intake for diabetes mellitus or arterial hypertension
- Medication or supplement intake with impact on immune function
- Previous or current alcohol and/or drug abuse or addiction

Precipitation of RNA

We performed an ethanol precipitation of RNA with ammonium acetate in order to reduce the volume of eluted RNA to 12 µl. The procedure was based on a published protocol on ethanol precipitation of RNA [70] as well as on internal laboratory modifications. Modifications are explained below. We added 0.5 volume of 7,5 M ammonium acetate and 4 volumes of 100% ethanol to the eluted RNA. Centrifugations were all carried out at 4 °C. The RNA pellet was washed twice. The RNA pellet was finally dissolved in 12 µl nuclease-free water by an incubation at 50 °C for 10 min in a thermoblock.
